# Oncogenes and tumor suppressor genes in squamous cell carcinoma of the tongue in young patients

**DOI:** 10.18632/oncotarget.2850

**Published:** 2015-01-30

**Authors:** Andreas Knopf, Justine Lempart, Murat Bas, Julia Slotta-Huspenina, Naglaa Mansour, Marie Kristin Fritsche

**Affiliations:** ^1^ Technische Universität München, Hals-Nasen-Ohrenklinik und Poliklinik, 81675 München, Germany; ^2^ Technische Universität München, Institut für Allgemeine Pathologie und Pathologische Anatomie, 81675 München, Germany

**Keywords:** Tongue, oncogenes, tumour suppressor genes, oral cancer, young patients

## Abstract

**Objectives:**

The occurrence of squamous cell carcinoma of the tongue (SCCT) of young patients increased. There are still controversies about patient prognosis. The underlying molecular mechanisms remain unclear.

**Methods:**

276 patients (66 ≤45, 210 >45 years) with SCCT were included. Clinical parameters and survival data were assessed. Oncogenes and tumor suppressors were analyzed via immunohistochemistry (p53, CXCR4, p16, EGFR) and qPCR (*CDK4, CDKN2A, TP53, MDM2, AKT1, PIK3CA, NRAS, HRAS, KRAS, HGF, MET, EGF, ATM, BRCA1, E2F1, FHIT, RUNX3, STK11, BCL2, CTNNB1*).

**Results:**

The median overall survival was 142 (≤45 years) and 34 months (>45 years) (*p* < 0.0001; HR [95%CI]: 0.37 [0.30–0.58]). Disease specific survival in patients ≤45 years was with 181 months significantly higher than in patients >45 years (*p* < 0.0001; HR [95%CI]: 0.33 [0.26–0.57]). Immunhistochemistry visualized a comparable expression of analyzed proteins. QPCR demonstrated in patients ≤45 years a higher expression of genes that are associated with carcinogenesis (*CTNNB1, STK11, CDKN2A, HGF, MET*) as well as tumor suppressors that constitute an enhanced radio-sensitivity (*ATM, BRCA1E2F1, FHIT*).

**Conclusion:**

Derogation of the *WNT-CTNNB1-STK11* and *CDKN2A-HGF-MET* pathway can constitute the carcinogenesis, while the higher expression of radio-sensitizers *ATM, BRCA1E2F1 and FHIT* can explain the better OS/DSS in young patients.

## INTRODUCTION

Head and neck squamous cell carcinoma (HNSCC) is the sixth most common malignancy worldwide with an annual incidence of more than 270,000 cases [1]. The 5-year survival rate is estimated to be about 50% [2]. While the overall incidence rate in HNSCC slightly reduced over the past decade, the occurrence of oral HNSCC increased [3–8]. Particularly, the incidence rate of young (<45 years) non-smokers with squamous cell carcinoma of the tongue (SCCT) increased significantly [8–11]. These patients show a highly aggressive phenotype with a high proportion of lymph node positivity at the time of diagnosis, a high recurrence rate after therapy, and therefore a poor prognosis [8, 12, 13]. The molecular mechanisms underlying the clinical behavior remain unclear. In common oral HNSCC alcohol and nicotine abuse represent the most important risk factors [7, 14–16]. Recently, the association between human papilloma virus (HPV) and tongue cancer (SCCT) was demonstrated with a HPV-positivity in 25–60% of SCCT [7]. Saito et al. observed an increased incidence of HPV-positive SCCT in Japan [15]. Recent studies failed to demonstrate an HPV-association in SCCT of young patients [3, 10]. Genomic aberrations apart from alcohol, nicotine, or virus induced carcinogenesis might constitute the malignant phenotype [17–19]. The activation of oncogenes (*EGFR, CCDN1, MYC, PIK3CA, RAS)* and an abrogated pathway in tumour suppressors (*TP53, TP73, RB, CDKN2A*, *CDKN1A)* could be widely demonstrated in HNSCC [20–28]. The purpose of this study is to give a detailed assessment of the oncogene and tumor suppressor profile in patients with common SCCT (>45 years) compared with SCCT in younger patients (≤45 years) using quantitative PCR (qPCR) and EGFR, p53, p16, and CXCR4 immunohistochemistry.

## RESULTS

### Epidemiology

From January 1983 to December 2013, a total of 276 patients with SCCT were treated in the Department of Otorhinolaryngology, Technical University Munich. There were 66 patients ≤45 years (mean: 38 years; range: 20–45 years) and 210 patients >45 years (mean: 60 years; range: 46–91 years). Both groups demonstrated the same gender distribution resulting in a moderate male predominance of 2.7 and 2.8 to 1. We did not see any differences in the nicotine and alcohol consumption (*p* = 0.41; *p* = 0.22). The cumulative consumption differed significantly. The prevalence of synchronous and metachronous malignancy was 24% in patients ≤45 years and 31% in patients >45 years (*p* = 0.3). In synchronous and metachronous malignancy pharyngeal carcinoma occurred in 9%, bronchial carcinoma in 7%, urogenital carcinoma in 5%, oesophageal carcinoma in 4%, oral cavity carcinoma (other than tongue) in 3%, gastro-intestinal carcinoma 3%, laryngeal carcinoma in 2%, cutaneous carcinoma in 2%, and other malignancies in 3% respectively. With respect to the TNM-stage, patients ≤45 years demonstrated significant smaller tumour sizes (*p* = 0.011), while the N- and M-status was comparable in both groups (*p* = 0.59; *p* = 0.76). The majority of patients were diagnosed with T1/2 tumours, solitary neck metastases (N1/2a), moderate histological differentiation (G2) and histologically complete resection (R0). Thirty-two patients (12%) underwent a primary radio-(chemo-) therapy. The majority of patients (68%) underwent radical surgical approaches comprising the trans-oral resection, mandible split with subsequent reconstruction, and neck dissection. An adjuvant radio-(chemo-) therapy was done in 111 patients (45%).

### Survival analysis

**Figure 1:**
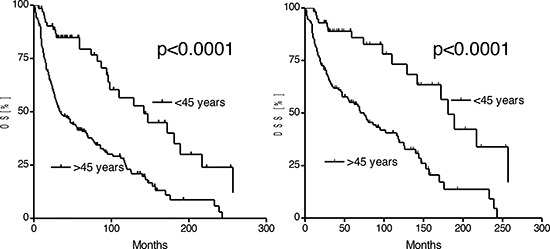
Kaplan-Meier estimates of the overall (OS) and disease specific survival in patients ≤45 years and patients >45 years

Recurrent disease was demonstrated in 20% of patients ≤45 years and 18% of patients >45 years (*p* = 0.3). The 5-year recurrence free interval (RFI) in patients ≤45 years was 84%, in patients >45 years 75% respectively. After a mean follow-up of 54 months in both, patients ≤45 years and >45 years, there were significant differences in the OS and DSS. The median OS in patients ≤45 years was 142 months and 34 months in patients >45 years, respectively (*p* < 0.0001; HR [95%CI]: 0.37 [0.30–0.58]). DSS in patients ≤45 years was with 181 months significantly higher than in patients >45 years (*p* < 0.0001; HR [95%CI]: 0.33 [0.26–0.57]). The 5-year survival rate in patients ≤45 years was 79%, in patients >45 years 41% (Fig. 1).

### Immunohistochemistry

**Figure 2:**
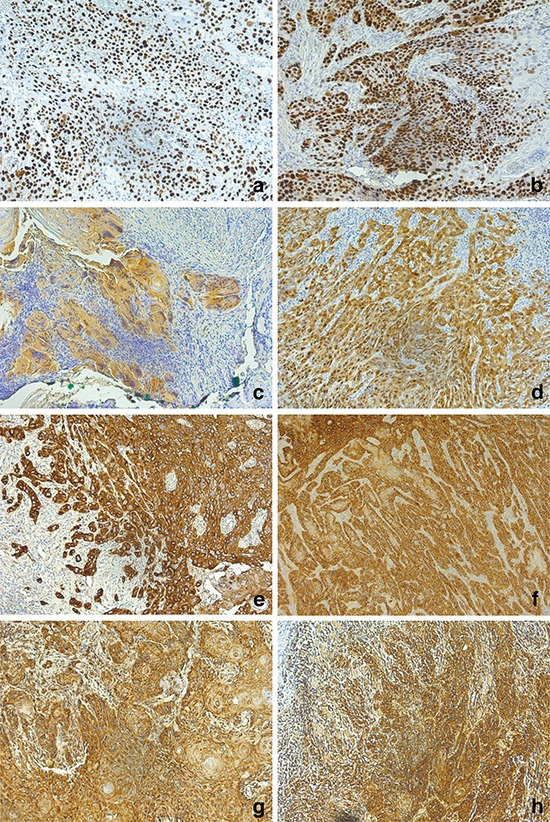
Immunohistochemistry (20x) of p53 (a, b), p16 (c, d), EGFR (e, f), and CXCR4 (g, h) Tissue samples of the cohort of patients ≤45 years are visualized on the left, the cohort of patients >45 years on the.

Immunohistochemistry did not reveal any differences between both groups. 73% of the patients ≤45 years and 87% of patients >45 years demonstrated p53-positivity (Fig. 2a, 2b). A positive p16 staining, as visualized in Fig. 2c and 2d, was seen in 13% of the patients ≤45 years and in 33% of patients >45 years. The vast majority of patients were p16 negative. All analyzed samples showed a strong EGFR and CXCR4 staining (Fig. 2e–h).

### Quantitative PCR of oncogenes and tumor suppressors

**Figure 3:**
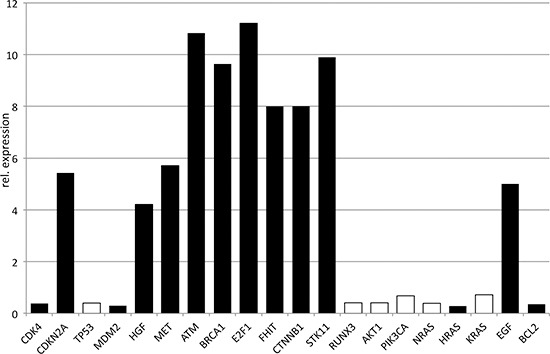
Quantitative PCR of oncogenes and tumor suppressors The relative expression is visualized for patients ≤45 years, normalized by patients >45 years. Significant differences were marked black.

*RUNX3, AKT1, PIK3CA,* the *RAS* family, *TP53, MDM2, CDK4*, and *BCL2* demonstrated comparable expression levels or were slightly minor expressed in patients ≤45 years (Fig. 3). *CDKN2A, MET, HGF*, *ATM, BRCA1, E2F1, FHIT, CTNNB1,* and *STK11* were significantly higher expressed in patients ≤45 years demonstrating differences from 4- to 11-fold (Fig. 3).

## DISCUSSION

Squamous cell carcinoma represents the most frequent histological differentiation in head and neck malignancy. While overall incidence rates decrease, several studies demonstrated an increased incidence of oral tongue squamous cell carcinomas (SCCT) in young adults [6, 10, 11, 29–31]. Many authors attribute SCCT of young adults a more aggressive phenotype with a high proportion of lymph node positivity at the time of diagnosis and a high recurrence rate after therapy. But, there are still controversies about the patient prognosis [8, 12, 13, 32–40]. The molecular mechanisms underlying the aggressive behavior remain unclear. The heterogeneity of head and neck squamous cell carcinoma was demonstrated in whole-exome sequencing identifying a small number of activating mutations in oncogenes and highlighting the role of *p53, Rb/INK4/ARF* and *Notch* tumor suppressor pathways [18, 21, 22, 41]. *EGFR* represents an oncogene that is frequently over-expressed in HNSCC due to activating mutations. After binding of EGF to its receptor the dimerization of EGFR results in an auto-phosphorylation of several tyrosine residues that lead to cell proliferation by activating the *MAPK*, *AKT*, and *JNK* pathways. In the current study, the majority of samples stained positive for EGFR without differences between the subgroups. While qPCR of *AKT1* did not reveal any differences between the groups, *EGF* was significantly higher expressed in young adults. In-vitro analysis demonstrated a dose-dependent rise of BCL-x(L) and p21(CIP1/WAF1) protein after incubation with EGF, whereas no influence was seen on BCL-2 [42]. According with the recent literature, qPCR showed a slight minor-expression of *BCL-2* in patients ≤45 years, despite *EGF* over-expression. Thus, a disruption of *the EGF-EGFR-AKT1* pathway that mediates the hypothesized aggressive phenotype of SCCT in young adults might be unlikely. *PIK3CA*, the *RAS*-family, *RUNX3*, or CXCR4 that were associated with loco-regional metastases when hyper-methylated or over-expressed showed a comparable or minor expression in patients ≤45 years. In contrast with recently published data, in our cohort *CTNNB1* expression did not correlate with tumor differentiation or lymph node metastasis. Authors notice that *CTNNB1* expression was no independent prognostic factor for disease-specific survival [43]. The *WNT/CTNNB1* pathway inhibits mitochondria- and detachment-mediated apoptosis [44]. *In vitro* analysis demonstrated that *CTNNB1* silencing activates the *STK11/AMPK* pathway resulting in a G1 arrest by phosphorylating p53 and suppressing *mTOR* signaling [45]. In our cohort, *STK11* was significantly higher expressed in patients ≤45 years. Besides *STK11, TP53* represents a further tumor suppressor that is frequently mutated in SCCT [46]. The *TP53-CDKN2A-CCND1-CDK4-RB1* pathway represents a tumor suppressor pathway that is frequently abrogated in HNSCC. Immunhistochemistry and qPCR identified p53 positivity in the majority of samples without any differences between the subgroups. QPCR revealed a higher expression of *CDKN2A* in patients ≤45 years, while *CDK4* was down-regulated. Subsequent p16 protein was visualized in 13% of patients ≤45 years and 33% of patients >45 years, indicating a minor impact of HPV associated carcinogenesis in the group of young adults. Infection with high-risk HPV is recognized as an independent risk factor, particularly in oropharyngeal tumor sites. HPV-positive patients with oropharyngeal carcinomas demonstrate a better survival, irrespective of the treatment regimen. HPV associated carcinogenesis in oral carcinomas has been discussed controversially so far [47, 48]. Beside a HPV induced p16 over-expression, abrogation of the *CDKN2A-CCND1-CDK4-RB1* pathway is a frequent event in head and neck squamous cell carcinoma. In our cohort, the *CDKN2A* over-expression in the absence of a p16 protein refers most likely to *CDKN2A* deficiency. Mutation, hypermethylation, and allelic alteration (loss of heterozygosity (LOH), microsatellite size alteration) of *CKDN2A* were associated with the development of dysplastic lesions [22, 49–52]. Particularly, LOH and absence of p16 protein might be associated with the progression of normal mucosa to hyperplastic lesion or carcinoma in situ [49, 53, 54]. Recently, in malignant melanoma a *CDKN2A* deficient mouse cell line demonstrated *MET* gene amplification [55]. The *HGF-MET* pathway plays a pivotal role in the progression of head and neck squamous cell carcinoma [56]. Particularly HPV negative tonsillar carcinomas with *HGF* or *MET* over-expression where associated with a reduced survival [46]. In our cohort, we identified an *MET-HGF* over-expression in patients ≤45 years.

Contradictory results in survival data might refer to different study populations and treatment regimens. In agreement with the present literature, we observed a high incidence of T1/2 tumors and solitary neck metastases [8, 12, 45]. Radical and subsequent reconstructive surgery resulted in a R0-resection the majority of patients. 111 patients (45%) underwent an adjuvant radio-(chemo-) therapy. We achieved a significant better loco-regional control with a 5-year RFI of 84% in patients ≤45 years and 75% in patients >45 years respectively. Other studies with a comparable study population and treatment regimen also demonstrated a significant higher OS and DSS in patients ≤45 years when compared with their older counterparts [6, 8, 12]. An enhanced radiosensitivity can constitute the better survival in our cohort. Tumor suppressors *ATM, BRCA1*, and *E2F1* were significantly higher expressed in patients ≤45 years. *ATM* plays a pivotal role in the homologous recombination after DNA double strand breaks. ATM or ATR kinases phosphorylate and therefore stabilize E2F1 transcription factor. *E2F1* represents a member of the *E2F* family that exclusively interacts with the *Rb* tumor suppressor. Furthermore, *E2F1* regulates *p53* dependent apoptosis [57]. Pusapati et al. demonstrated an accelerated *Myc* induced tumorigenesis in the oral cavity in *E2F1* deficient mice [58]. Wang et al. established the association of *BRCA1* with *ATM* and DNA repair proteins in the “BRCA1-associated genome surveillance complex (BASC)” [59]. The derogation of the *ATM-ATR-CHEK1* pathway was associated with radioresistance in HNSCC [60, 61]. *FHIT* represents a further tumor suppressor gene that was associated with patients' prognosis in oral squamous cell carcinoma. *FHIT* negativity correlated with cervical lymph node metastasis and poor disease-specific survival [62, 63]. FHIT interacts with the *CHEK1* pathway in an opposing manner. Fhit negative cells demonstrate an over-activated *ATM-ATR-CHEK1* pathway that is associated with an increased mutation frequency and therefore disturbed functional integrity [64]. QPCR revealed an 8-fold higher expression of *FHIT* in young adults. The higher expression of *ATM, BRCA1*, *E2F1, and FHIT* in patients ≤45 years can result in an enhanced radio-sensitivity can and therefore better survival in our cohort.

## CONCLUSION

The derogation of the *WNT-CTNNB1-STK11* and *CDKN2A-HGF-MET* pathway can constitute the carcinogenesis in young patients with SCCT where a longstanding nicotine or alcohol abuse is missing. The higher expression of *ATM, BRCA1*, *E2F1 and FHIT* and subsequent enhanced radios-sensitivity can explain the better OS/DSS in patients ≤45 years.

## MATERIALS AND METHODS

### Patient selection

The study included 66 patients with SCCT ≤45 years and 210 patients >45 years. SCCT tumor samples were histologically reviewed by at least two experienced pathologists. Dysplasia, carcinoma in situ and other histologic subtypes were excluded. Clinical parameters and survival data were retrospectively collected including age, sex, alcohol and nicotine abuse, TNM-staging, grading, treatment modalities, recurrence and death/loss to follow-up. Patients with lacking data, incomplete staging, and refused/not finished treatment were excluded from survival analysis. The mean follow-up time was 54 months [range: 0–301 months]. Paraffin-embedded tumor (FFPE) samples from 15 SCCT patients ≤45 years and 15 patients >45 years were randomly selected and analyzed in quantitative PCR (qPCR) and immunohistochemistry (IHC).

### Statistical analysis

Differences between both groups were analyzed using the Chi square test and Fisher exact test for categorical, and the unpaired student's t-test for continuous variables. As main endpoints the overall survival (OS), disease-specific survival (DSS) and recurrence-free interval (RFI) were assessed measuring the time from treatment to death of any cause, tumor-related death and loco-regional recurrence, and/or distant metastasis. Survival rates and curves were calculated and illustrated by the Kaplan-Meier method and further analyzed by the log-rank test for univariate analysis. *P*-values < 0.05 were considered statistically significant (Graph Pad Prism, La Jolla, USA).

### Immunohistochemistry

FFPE tumor sections (2.5 μm) were p16 (Ventana, Tuscon, USA, ready to use), p53 (Do-7, Dako, Hamburg, Germany, 1:200), EGFR (US Biological, Hamburg, Germany, 1:200), and CXCR4 (R&D, Wiesbaden, Germany, 1:200) stained and visualized with the Bond Polymer Refine Detection Kit (Leica, Nussloch, Germany). Expression levels were classified using a scoring system analyzing the staining intensity (0 = no staining, 1 = low, 2 = moderate, 3 = strong staining intensity) and the relative proportion of stained cells (0, 1 =< 10%, 2 = 10–39%, 3 = 40–69%, 4 => 70 of the tumor cells). A cumulative score (range 0–7 points) was assessed by adding both scores. A positive staining was defined by a cumulative score equal or greater than 3.

### RNA extraction and quantitative PCR

Tumor areas were identified after hematoxylin staining and corresponding areas micro-dissected from untreated tumor sections (10 μm). RNA extraction was done according to the manufacturer's protocol, including a DNase digestion (FFPE RNA Micro Kit, Roche, Mannheim, Germany). RNA concentration and purity was determined using a Nanodrop system (Thermo Scientific, Wilmington, USA). 250 ng of total RNA was reverse transcribed and pre-amplified using the RT^2^ FFPE PreAMP cDNA Synthesis Kit (Qiagen, Hilden, Germany). Subsequent qPCR was performed using the RT^2^ qPCR Master Mix and the RT^2^ Profiler™ PCR Array for Oncogenes and Tumor Suppressor (*CDK4, CDKN2A, TP53, MDM2, AKT1, PIK3CA, NRAS, HRAS, KRAS, HGF, MET, EGF, ATM, BRCA1, E2F1, FHIT, RUNX3, STK11, BCL2,* and *CTNNB1*) (Qiagen). Gene-specific PCR products were continuously measured during 40 cycles with the BioRad D-CFX96 Cycler (München, Germany). Results were evaluated using the 2-ΔΔCT method. Data analysis was done using the RT^2^ Profiler PCR Array Data Analysis version 3.5 (Qiagen). Target gene expression was normalized between different samples based on the values of *GAPDH* expression. A fold difference >2.5 was considered statistically significant.

## References

[1] Parkin DM, Bray F, Ferlay J, Pisani P (2005). Global cancer statistics, 2002. CA Cancer J Clin.

[2] Fuller CD, Wang SJ, Thomas CR, Hoffman HT, Weber RS, Rosenthal DI (2007). Conditional survival in head and neck squamous cell carcinoma: results from the SEER dataset 1973–1998. Cancer.

[3] Harris SL, Kimple RJ, Hayes DN, Couch ME, Rosenman JG (2010). Never-smokers, never-drinkers: unique clinical subgroup of young patients with head and neck squamous cell cancers. Head Neck.

[4] Chaturvedi AK, Engels EA, Anderson WF, Gillison ML (2008). Incidence trends for human papillomavirus-related and -unrelated oral squamous cell carcinomas in the United States. J Clin Oncol.

[5] Golas SM (2007). Trends in palatine tonsillar cancer incidence and mortality rates in the United States. Community Dent Oral Epidemiol.

[6] Annertz K, Anderson H, Biorklund A, Moller T, Kantola S, Mork J, Olsen JH, Wennerberg J (2002). Incidence and survival of squamous cell carcinoma of the tongue in Scandinavia, with special reference to young adults. Int J Cancer.

[7] Wittekindt C, Wagner S, Mayer CS, Klussmann JP (2012). Basics of tumor development and importance of human papilloma virus (HPV) for head and neck cancer. GMS Curr Top Otorhinolaryngol Head Neck Surg.

[8] Shiboski CH, Schmidt BL, Jordan RC (2005). Tongue and tonsil carcinoma: increasing trends in the U.S. population ages 20–44 years. Cancer.

[9] Catania JA, Osmond D, Neilands TB, Canchola J, Gregorich S, Shiboski S (2005). Commentary on Schroder, et al (2003a, 2003b). Ann Behav Med.

[10] Patel SC, Carpenter WR, Tyree S, Couch ME, Weissler M, Hackman T, Hayes DN, Shores C, Chera BS (2011). Increasing incidence of oral tongue squamous cell carcinoma in young white women, age 18 to 44 years. J Clin Oncol.

[11] Myers JN, Elkins T, Roberts D, Byers RM (2000). Squamous cell carcinoma of the tongue in young adults: increasing incidence and factors that predict treatment outcomes. Otolaryngol Head Neck Surg.

[12] Manuel S, Raghavan SK, Pandey M, Sebastian P (2003). Survival in patients under 45 years with squamous cell carcinoma of the oral tongue. Int J Oral Maxillofac Surg.

[13] Llewellyn CD, Linklater K, Bell J, Johnson NW, Warnakulasuriya KA (2003). Squamous cell carcinoma of the oral cavity in patients aged 45 years and under: a descriptive analysis of 116 cases diagnosed in the South East of England from 1990 to 1997. Oral Oncol.

[14] Chen KM, Guttenplan JB, Zhang SM, Aliaga C, Cooper TK, Sun YW, DelTondo J, Kosinska W, Sharma AK, Jiang K (2013). Mechanisms of oral carcinogenesis induced by dibenzo[a,l]pyrene: an environmental pollutant and a tobacco smoke constituent. Int J Cancer.

[15] Saito Y, Yoshida M, Ushiku T, Omura G, Ebihara Y, Shimono T, Fukayama M, Yamasoba T, Asakage T (2013). Prognostic value of p16 expression and alcohol consumption in Japanese patients with oropharyngeal squamous cell carcinoma. Cancer.

[16] Sturgis EM, Wei Q, Spitz MR (2004). Descriptive epidemiology and risk factors for head and neck cancer. Semin Oncol.

[17] van Monsjou HS, Lopez-Yurda MI, Hauptmann M, van den Brekel MW, Balm AJ, Wreesmann VB (2013). Oral and oropharyngeal squamous cell carcinoma in young patients: the Netherlands Cancer Institute experience. Head Neck.

[18] Stransky N, Egloff AM, Tward AD, Kostic AD, Cibulskis K, Sivachenko A, Kryukov GV, Lawrence MS, Sougnez C, McKenna A (2011). The mutational landscape of head and neck squamous cell carcinoma. Science.

[19] Agrawal A, Rao KS, Makannavar JH, Shetty L, Patel N (2007). Extracranial meningioma in the vicinity of the temporal bone: a difficult preoperative diagnosis. Surg Neurol.

[20] Yamazaki Y, Chiba I, Hirai A, Sugiura C, Notani K, Kashiwazaki H, Tei K, Totsuka Y, Fukuda H (2003). Specific p53 mutations predict poor prognosis in oral squamous cell carcinoma. Oral Oncol.

[21] Agrawal N, Frederick MJ, Pickering CR, Bettegowda C, Chang K, Li RJ, Fakhry C, Xie TX, Zhang J, Wang J (2011). Exome sequencing of head and neck squamous cell carcinoma reveals inactivating mutations in NOTCH1. Science.

[22] Rothenberg SM, Ellisen LW (2012). The molecular pathogenesis of head and neck squamous cell carcinoma. J Clin Invest.

[23] Perez-Ordonez B, Beauchemin M, Jordan RC (2006). Molecular biology of squamous cell carcinoma of the head and neck. J Clin Pathol.

[24] Sakai E, Rikimaru K, Ueda M, Matsumoto Y, Ishii N, Enomoto S, Yamamoto H, Tsuchida N (1992). The p53 tumor-suppressor gene and ras oncogene mutations in oral squamous-cell carcinoma. Int J Cancer.

[25] Kastan MB, Zhan Q, el-Deiry WS, Carrier F, Jacks T, Walsh WV, Plunkett BS, Vogelstein B, Fornace AJ (1992). A mammalian cell cycle checkpoint pathway utilizing p53 and GADD45 is defective in ataxia-telangiectasia. Cell.

[26] Barak Y, Juven T, Haffner R, Oren M (1993). mdm2 expression is induced by wild type p53 activity. EMBO J.

[27] Reed AL, Califano J, Cairns P, Westra WH, Jones RM, Koch W, Ahrendt S, Eby Y, Sewell D, Nawroz H (1996). High frequency of p16 (CDKN2/MTS-1/INK4A) inactivation in head and neck squamous cell carcinoma. Cancer Res.

[28] Harbour JW, Luo RX, Dei SA, Postigo AA, Dean DC (1999). Cdk phosphorylation triggers sequential intramolecular interactions that progressively block Rb functions as cells move through G1. Cell.

[29] Davis S, Severson RK (1987). Increasing incidence of cancer of the tongue in the United States among young adults. Lancet.

[30] Schantz SP, Byers RM, Goepfert H (1988). Tobacco and cancer of the tongue in young adults. JAMA.

[31] Brown LM, Check DP, Devesa SS (2012). Oral cavity and pharynx cancer incidence trends by subsite in the United States: changing gender patterns. J Oncol.

[32] Depue RH (1986). Rising mortality from cancer of the tongue in young white males. N Engl J Med.

[33] Sarkaria JN, Harari PM (1994). Oral tongue cancer in young adults less than 40 years of age: rationale for aggressive therapy. Head Neck.

[34] Atula S, Grenman R, Laippala P, Syrjanen S (1996). Cancer of the tongue in patients younger than 40 years. A distinct entity?. Arch Otolaryngol Head Neck Surg.

[35] Friedlander PL, Schantz SP, Shaha AR, Yu G, Shah JP (1998). Squamous cell carcinoma of the tongue in young patients: a matched-pair analysis. Head Neck.

[36] Lund VJ, Howard DJ (1990). Head and neck cancer in the young: a prognostic conundrum?. J Laryngol Otol.

[37] Randall CJ, Shaw HJ (1986). Malignant tumours of the tongue in young adults. Experience of a secondary referral centre. J Laryngol Otol.

[38] Siegelmann-Danieli N, Hanlon A, Ridge JA, Padmore R, Fein DA, Langer CJ (1998). Oral tongue cancer in patients less than 45 years old: institutional experience and comparison with older patients. J Clin Oncol.

[39] Verschuur HP, Irish JC, O'sullivan B, Goh C, Gullane PJ, Pintilie M (1999). A matched control study of treatment outcome in young patients with squamous cell carcinoma of the head and neck. Laryngoscope.

[40] Clarke RW, Stell PM (1992). Squamous carcinoma of the head and neck in the young adult. Clin Otolaryngol Allied Sci.

[41] De Carvalho TG, De Carvalho AC, Maia DC, Ogawa JK, Carvalho AL, Vettore AL (2013). Search for mutations in signaling pathways in head and neck squamous cell carcinoma. Oncol Rep.

[42] Ruddel J, Wennekes VE, Meissner W, Werner JA, Mandic R (2010). EGF-dependent induction of BCL-xL and p21CIP1/WAF1 is highly variable in HNSCC cells--implications for EGFR-targeted therapies. Anticancer Res.

[43] Rosado P, Lequerica-Fernandez P, Fernandez S, Allonca E, Villallain L, de Vicente JC (2013). E-cadherin and beta-catenin expression in well-differentiated and moderately-differentiated oral squamous cell carcinoma: relations with clinical variables. Br J Oral Maxillofac Surg.

[44] Gao C, Cao W, Bao L, Zuo W, Xie G, Cai T, Fu W, Zhang J, Wu W, Zhang X (2010). Autophagy negatively regulates Wnt signalling by promoting Dishevelled degradation. Nat Cell Biol.

[45] Chang HW, Lee YS, Nam HY, Han MW, Kim HJ, Moon SY, Jeon H, Park JJ, Carey TE, Chang SE (2013). Knockdown of beta-catenin controls both apoptotic and autophagic cell death through LKB1/AMPK signaling in head and neck squamous cell carcinoma cell lines. Cell Signal.

[46] Tan DS, Wang W, Leong HS, Sew PH, Lau DP, Chong FT, Krisna SS, Lim TK, Iyer NG (2014). Tongue carcinoma infrequently harbor common actionable genetic alterations. BMC Cancer.

[47] El-Mofty SK, Lu DW (2003). Prevalence of human papillomavirus type 16 DNA in squamous cell carcinoma of the palatine tonsil, and not the oral cavity, in young patients: a distinct clinicopathologic and molecular disease entity. Am J Surg Pathol.

[48] Woods KV, Shillitoe EJ, Spitz MR, Schantz SP, Adler-Storthz K (1993). Analysis of human papillomavirus DNA in oral squamous cell carcinomas. J Oral Pathol Med.

[49] Tripathi A, Banerjee S, Roy A, Roychowdhury S, Panda CK (2003). Alterations of the P16 gene in uterine cervical carcinoma from Indian patients. Int J Gynecol Cancer.

[50] Lin SC, Chang KW, Chang CS, Liu TY, Tzeng YS, Yang FS, Wong YK (2000). Alterations of p16/MTS1 gene in oral squamous cell carcinomas from Taiwanese. J Oral Pathol Med.

[51] Zhang SY, Klein-Szanto AJ, Sauter ER, Shafarenko M, Mitsunaga S, Nobori T, Carson DA, Ridge JA, Goodrow TL (1994). Higher frequency of alterations in the p16/CDKN2 gene in squamous cell carcinoma cell lines than in primary tumors of the head and neck. Cancer Res.

[52] Wu CL, Roz L, McKown S, Sloan P, Read AP, Holland S, Porter S, Scully C, Paterson I, Tavassoli M (1999). DNA studies underestimate the major role of CDKN2A inactivation in oral and oropharyngeal squamous cell carcinomas. Genes Chromosomes Cancer.

[53] El-Naggar AK, Lai S, Clayman GL, Zhou JH, Tucker SA, Myers J, Luna MA, Benedict WF (1999). Expression of p16, Rb, and cyclin D1 gene products in oral and laryngeal squamous carcinoma: biological and clinical implications. Hum Pathol.

[54] Nagai MA (1999). Genetic alterations in head and neck squamous cell carcinomas. Braz J Med Biol Res.

[55] Vanbrocklin MW, Robinson JP, Whitwam T, Guilbeault AR, Koeman J, Swiatek PJ, Vande Woude GF, Khoury JD, Holmen SL (2009). Met amplification and tumor progression in Cdkn2a-deficient melanocytes. Pigment Cell Melanoma Res.

[56] Lau PC, Chan AT (2011). Novel therapeutic target for head and neck squamous cell carcinoma: HGF-MET signaling pathway. Anticancer Drugs.

[57] Polager S, Ginsberg D (2008). Trends Cell Biol.

[58] Pusapati RV, Weaks RL, Rounbehler RJ, McArthur MJ, Johnson DG (2010). E2F2 suppresses Myc-induced proliferation and tumorigenesis. Mol Carcinog.

[59] Wang Y, Cortez D, Yazdi P, Neff N, Elledge SJ, Qin J (2000). BASC, a super complex of BRCA1-associated proteins involved in the recognition and repair of aberrant DNA structures. Genes Dev.

[60] Sankunny M, Parikh RA, Lewis DW, Gooding WE, Saunders WS, Gollin SM (2014). Targeted inhibition of ATR or CHEK1 reverses radioresistance in oral squamous cell carcinoma cells with distal chromosome arm 11q loss. Genes Chromosomes Cancer.

[61] Mansour WY, Bogdanova NV, Kasten-Pisula U, Rieckmann T, Kocher S, Borgmann K, Baumann M, Krause M, Petersen C, Hu H (2013). Aberrant overexpression of miR-421 downregulates ATM and leads to a pronounced DSB repair defect and clinical hypersensitivity in SKX squamous cell carcinoma. Radiother Oncol.

[62] Joo YH, Park SW, Jung SH, Lee YS, Nam IC, Cho KJ, Park JO, Chung YJ, Kim MS (2013). Recurrent loss of the FHIT gene and its impact on lymphatic metastasis in early oral squamous cell carcinoma. Acta Otolaryngol.

[63] Lee SH, Koo BS, Kim JM, Huang S, Rho YS, Bae WJ, Kang HJ, Kim YS, Moon JH, Lim YC (2014). Wnt/beta-catenin signalling maintains self-renewal and tumourigenicity of head and neck squamous cell carcinoma stem-like cells by activating Oct4. J Pathol.

[64] Hu B, Wang H, Wang X, Lu HR, Huang C, Powell SN, Huebner K, Wang Y (2005). Fhit and CHK1 have opposing effects on homologous recombination repair. Cancer Res.

